# Ovarian Mucinous Cystadenofibroma Mimicking Malignancy: A Case Report of a Rare Subtype

**DOI:** 10.7759/cureus.99827

**Published:** 2025-12-22

**Authors:** Sanghamitra Jena, R Krishna Gopal, Neetesh K Sinha, Anil Prasad, Jasmine Mallik

**Affiliations:** 1 Surgical Oncology, Tata Main Hospital, Jamshedpur, IND; 2 Pathology, Tata Main Hospital, Jamshedpur, IND

**Keywords:** benign, cystadenofibroma, differential diagnosis, frozen section, mucinous, ovarian tumors, ovary

## Abstract

Benign mucinous cystadenofibromas are very rare tumors of the ovary. They often mimic malignant pelvic tumors in terms of size and appearance. Here, we report a case of a 34-year-old female patient presenting with a 20 × 18 cm large abdominal mass. Clinically and radiologically, a diagnosis of a borderline malignant ovarian mass was made, and an exploratory laparotomy was planned. Intraoperative frozen section evaluation resulted in a diagnosis of benign mucinous cystadenofibroma. This highlights the need to consider mucinous cystadenofibroma as a differential diagnosis for large pelvic tumors and emphasizes the role of frozen section in the optimal surgical management of such cases.

## Introduction

Ovarian cystadenofibroma accounts for about 1.7% of all ovarian neoplasms [1]. The incidence of mucinous cystadenofibroma is even rarer. These tumors are epithelial in origin, comprising cystic and solid fibrotic components. Histologically, they can be classified as serous, mucinous, endometrioid, clear cell, or mixed categories [2]. All subtypes contain varying amounts of fibrous stroma. The presence of solid components or irregular thick septa on pre-operative imaging often makes differentiation from malignant neoplasms difficult [3]. On gross examination at the time of surgery, the lesion may resemble a malignant tumor. Therefore, frozen section plays a vital role in avoiding unnecessary extensive surgery for a benign mass in young female patients [2].

## Case presentation

A 34-year-old woman was evaluated in the Department of Surgical Oncology for a progressively enlarging abdominal swelling. She reported that the mass had increased noticeably over the previous five months and was accompanied by a persistent, dull abdominal discomfort. Her obstetric history included two vaginal deliveries. Her body mass index was 25. She had no known comorbidities and was not exposed to diethylstilbestrol, oral contraception, or any other hormone replacement therapy.

On general examination, no abnormalities were noted. Abdominal examination revealed a large, firm-to-cystic mass measuring approximately 20 × 18 cm, corresponding to a 28-week uterine size. The mass extended superiorly toward the xiphisternum and demonstrated limited mobility. Pelvic examination showed a normal cervix with free bilateral fornices.

**Figure 1:**
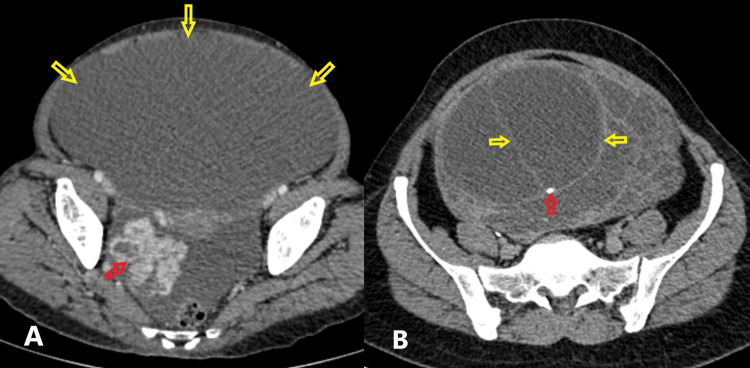
Contrast-Enhanced Computed Tomography (CECT) images A) CECT abdomen showing large showing large solid-cystic mass. The cystic component is marked with yellow arrows and solid component is marked with a red arrow; B) The mass has internal septations (marked with yellow arrows) and focal areas of calcification (marked with a red arrow).

Routine laboratory tests, including tumor markers (cancer antigen (CA)-125, 5.8 U/ml; beta-human chorionic gonadotropin (HCG), 0.6 mIU/ml; lactate dehydrogenase (LDH), 158 U/L; anti-mullerian hormone, 0.01 ng/ml; and alpha-fetoprotein, 1.66 ng/ml), were within normal ranges. An ultrasound of the abdomen detected a large cystic-solid space-occupying lesion (SOL) in the lower abdomen and pelvis with well-defined margins. Multiple thickened septations were present, some demonstrating internal vascularity with low-resistance flow. Certain compartments contained floating internal echoes suggestive of debris. Contrast-enhanced computed tomography (CECT) demonstrated a large solid-cystic mass situated close to both ureters, with several smooth, enhancing septa, raising concern for a possible borderline ovarian neoplasm. There were focal areas of calcification, and nodular enhancement was seen posteriorly in a few areas (Figure 1).

**Figure 2:**
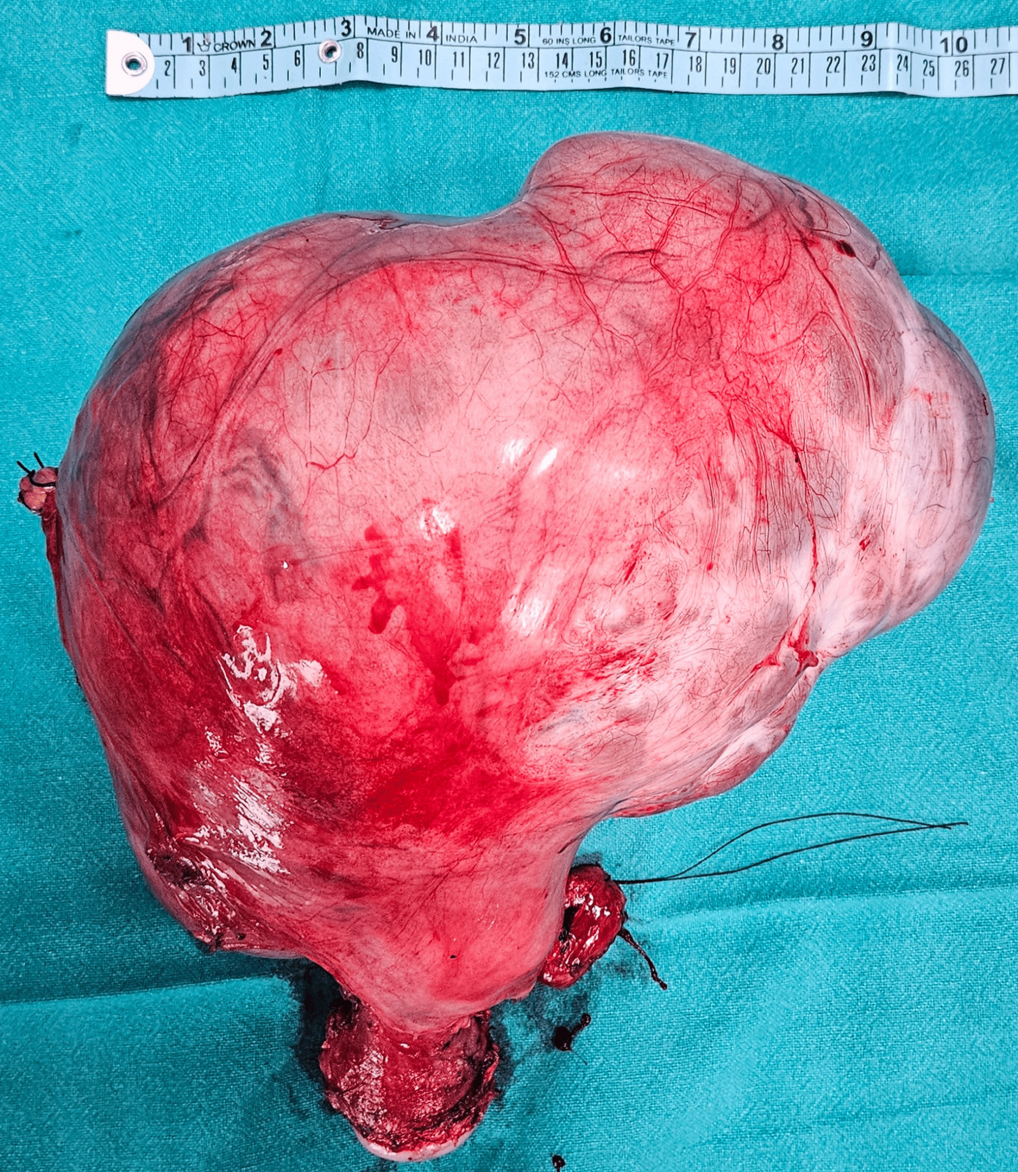
The resected TAH-BSO specimen TAH-BSO: total abdominal hysterectomy with bilateral salpingo-oophorectomy.

With a provisional impression of a benign to borderline right ovarian tumor, surgical exploration was planned. As the patient had completed childbearing, she elected to undergo total abdominal hysterectomy with bilateral salpingo-oophorectomy (TAH-BSO). Preoperative ureteric stents were inserted because of the mass’s size and anticipated surgical difficulty. Through a midline incision, a large abdominopelvic tumor presumed to originate from the right ovary was identified. It was densely adherent to the uterine body and fundus, and the right ureter coursed closely along its surface. The left adnexa appeared uninvolved. TAH-BSO was completed, and the specimen was submitted for intraoperative frozen-section evaluation. The resected mass measured 26 × 19 × 10 cm and weighed 3.6 kg (Figure 2).

**Figure 3:**
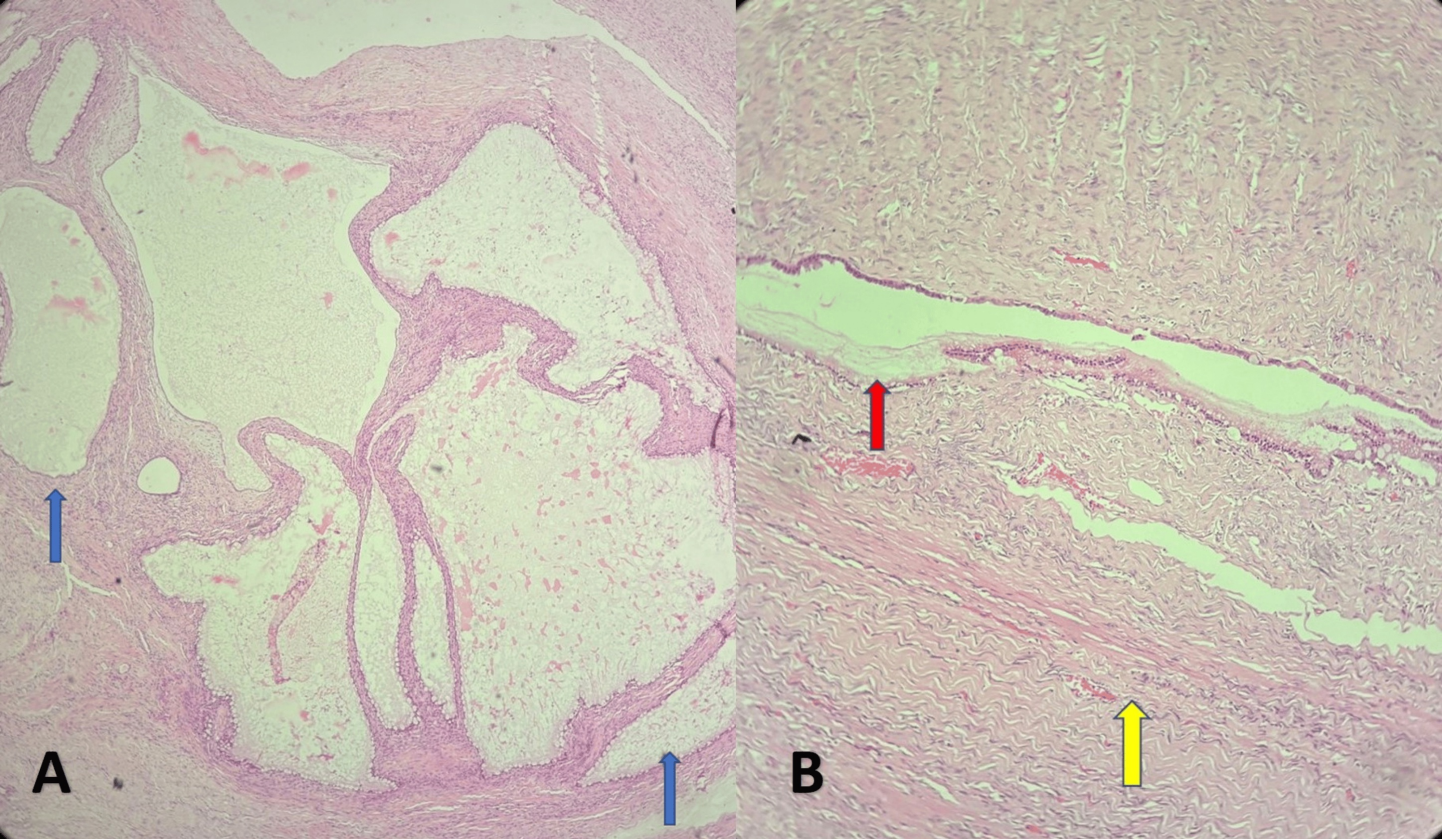
Microphotographs of the specimen A) Microphotograph showing multiloculated cyst lined by columnar epithelium (blue arrow) (40X H&E); B) Microphotograph showing fibrotic stroma (yellow arrow) and cyst with mucinous material (pink arrow) (100X H&E). H&E: Hematoxylin and Eosin.

The external surface of the mass was smooth and lobulated with an intact capsule. Sectioning released abundant thick, mucinous material. Numerous cystic compartments were present, with wall thickness varying between 1 and 3 mm. At some places, the cyst wall was thickened, forming solid areas. Microscopy revealed cystic cavities lined by intestinal-type columnar epithelium surrounded by dense fibrous stroma of monomorphic morphology. Foci of epithelial stratification up to three layers were noted. The epithelial cells had mild nuclear enlargement without nucleoli, and the proliferative epithelial component represented only about 5% of the tissue examined. No malignant features were detected. The frozen section diagnosis suggested a benign mucinous cystadenofibroma, which was confirmed on final histopathology (Figure 3).

The uterus, cervix, and left ovary were unremarkable.

The patient had an uncomplicated postoperative course and was discharged on the fifth postoperative day with advice to return for routine follow-up.

## Discussion

Ovarian cystadenofibromas are most commonly encountered in women during the reproductive and perimenopausal years, though they can occur across a broad age range [4]. Rare cases reported in younger patients have been linked to in-utero exposure to diethylstilbestrol [5]. Additional factors such as elevated body mass index and the use of hormone replacement therapy after menopause have been associated with a higher likelihood of developing these tumors [6].

Histologically, cystadenofibromas are categorized as benign, borderline, or malignant based on the extent of epithelial proliferation and how they interact with the supporting fibrous stroma. The vast majority fall into the benign category, with serous cystadenofibroma representing the most frequently encountered subtype [4,7].

Many individuals remain symptom-free, and the tumor is often discovered incidentally. When symptoms do occur, they typically relate to the mass effect, including lower abdominal or pelvic discomfort, a palpable mass, or pressure-related issues such as bowel or urinary disturbances. Less commonly, these tumors may lead to complications like torsion of the ovary, dysfunctional uterine bleeding, or ovulatory dysfunction [8,9].

Ultrasound findings can vary, but these lesions frequently present as cystic masses, either simple or multiloculated, with internal solid areas or papillary projections. About half of the cases demonstrate increased vascularity, which may complicate the distinction from malignant ovarian tumors [10]. CECT is likewise limited in reliably differentiating benign from malignant lesions due to overlapping appearances [1]. MRI, however, provides more specific diagnostic value. A characteristic feature is the presence of low-signal fibrous tissue interspersed with tiny, high-signal cystic spaces on T2-weighted imaging, creating a sponge-like pattern sometimes referred to as the “black sponge” sign [11,12]. In comparison, malignant counterparts such as cystadenocarcinofibromas typically show more prominent enhancement and higher T2 signal within the solid regions [3].

Surgical removal of the affected ovary is generally recommended. Because these tumors may resemble malignant masses during visual inspection, diagnostic laparoscopy offers limited benefit. Thorough pre-operative counselling helps set expectations regarding potential findings and management strategies [2,8]. 

Frozen section analysis during surgery plays an important role, as confirming benign pathology can prevent patients from unnecessarily undergoing extensive staging procedures [2]. In the literature search, frozen section has an overall accuracy of 91.85%. The sensitivity for benign, borderline, and malignant tumors is 99.2, 88.46, and 82.95% respectively, and the corresponding specificity is 96.5, 93.23, and 99.3% [13]. Diagnosing borderline mucinous and well-differentiated mucinous carcinoma is a challenge because of the large size of the tumor. The frozen section has constraints, like limited number of sections that can be examined, thicker sections compared to permanent sections, and freezing artifacts that obscure the finer details. Sampling errors can also occur because of the heterogeneity of the tumor [13]. In spite of these limitations, frozen section is an important diagnostic tool in the management of such cases.

## Conclusions

This case of a giant ovarian mucinous cystadenofibroma serves as a powerful illustration of several critical diagnostic and surgical challenges. This entity is a rare benign “masquerader” of malignancy. Its complex radiological features, including thick septations and internal vascularity, can create a convincing “false-positive” clinical impression of an advanced neoplasm. This case demonstrates that in the evaluation of giant ovarian masses, clinical assessment and imaging must guide surgical planning, as tumor markers are diagnostically unreliable. It also underscores the importance of meticulous pre-operative planning, including multidisciplinary collaboration such as involvement of the urologist and pathologist. An accurate frozen-section diagnosis was the pivotal factor that allowed safe and appropriate surgical de-escalation. This prevented the significant morbidity that could have resulted from an unnecessary full oncologic staging procedure, such as lymphadenectomy and omentectomy. So, it highlights the necessity for clinicians to recognize this rare diagnostic pitfall and to rely on a comprehensive, multidisciplinary management approach.
